# Nuclear targeted *Saccharomyces cerevisiae* asparagine synthetases associate with the mitotic spindle regardless of their enzymatic activity

**DOI:** 10.1371/journal.pone.0243742

**Published:** 2020-12-21

**Authors:** Chalongrat Noree, Naraporn Sirinonthanawech

**Affiliations:** Institute of Molecular Biosciences, Mahidol University, Salaya, Phuttamonthon, Nakhon Pathom, Thailand; CNR, ITALY

## Abstract

Recently, human asparagine synthetase has been found to be associated with the mitotic spindle. However, this event cannot be seen in yeast because yeast takes a different cell division process via closed mitosis (there is no nuclear envelope breakdown to allow the association between any cytosolic enzyme and mitotic spindle). To find out if yeast asparagine synthetase can also (but hiddenly) have this feature, the coding sequences of green fluorescent protein (*GFP*) and nuclear localization signal (*NLS*) were introduced downstream of *ASN1* and *ASN2*, encoding asparagine synthetases Asn1p and Asn2p, respectively, in the yeast genome having *mCherrry* coding sequence downstream of *TUB1* encoding alpha-tubulin, a building block of the mitotic spindle. The genomically engineered yeast strains showed co-localization of Asn1p-GFP-NLS (or Asn2p-GFP-NLS) and Tub1p-mCherry in dividing nuclei. In addition, an activity-disrupted mutation was introduced to *ASN1* (or *ASN2*). The yeast mutants still exhibited co-localization between defective asparagine synthetase and mitotic spindle, indicating that the biochemical activity of asparagine synthetase is not required for its association with the mitotic spindle. Furthermore, nocodazole treatment was used to depolymerize the mitotic spindle, resulting in lack of association between the enzyme and the mitotic spindle. Although yeast cell division undergoes closed mitosis, preventing the association of its asparagine synthetase with the mitotic spindle, however, by using yeast constructs with re-localized Asn1/2p have suggested the moonlighting role of asparagine synthetase in cell division of higher eukaryotes.

## Introduction

Several metabolic enzymes have been identified to be able to form visible intracellular structures in a variety of species, from bacteria to yeast, fly, and even mammals [1–10]. One of the most studied metabolic enzymes possessing self-assembly is CTP synthetase, the enzyme responsible for making CTP which is a key precursor for nucleic acid and lipid metabolism [11, 12]. It has been shown that supramolecular assembly of metabolic enzymes is utilized by living cells to modulate activities of those enzymes in response to the availability of enzyme substrates, products, and regulatory factors [1, 6–8, 13–18]. In bacteria and yeast, CTP synthetase assemblies comprise inactive form of the enzyme [13, 15]. But, in human cells, the active form of CTP synthetase is present in the assemblies [19], suggesting the diverse metabolic controls (on/off) through enzyme assembly in distinct species. Anyway, these findings have led to a proposition that the reversible assembly of a given metabolic enzyme acts as a supramolecular switch to stimulate (switch on) or to suppress (switch off) the enzyme activity and also direct the involved metabolic pathway(s), depending on metabolic states and cellular demands for the corresponding metabolites to support the relevant cellular process, cell growth and development at a certain period of time [8, 20–26].

Asparagine synthetase, catalyzing the ATP-dependent conversion of L-aspartate to L-asparagine [L-aspartate + L-glutamine + H_2_O + ATP → L-asparagine + L-glutamate + AMP + PP_i_], is another metabolic enzyme found to be able to reversibly form long filaments and foci in yeast cytoplasm [6, 9, 17, 27]. By introducing chromosomal mutations to the active sites within N-terminal and C-terminal domains, the activity of asparagine synthetase has been shown to be coupled to their supramolecular assembly/disassembly [17], providing another piece of evidence to help establish the current concept of reversible enzyme assembly as a biological event used for regulating the intracellular enzyme productivity. However, there is currently no report about the evolutionary conservation of asparagine synthetase assembly in the cytoplasm of mammalian cells. Unexpectedly, human asparagine synthetase (hASNS) has recently been found to cluster around centrosomes and line up with the mitotic spindles in actively dividing cells. Changes in the relative expression of hASNS to alpha-tubulin (a building block of mitotic spindle) when treating human cell lines with nocodazole (microtubule/mitotic spindle destabilizing agent) or asparaginase (medically used to lower intracellular asparagine levels, thus enhancing the expression of asparagine synthetase in order to maintain the amino acid homeostasis) have hinted a moonlighting function of human asparagine synthetase in cell division, in addition to its typical role in the amino acid metabolism [28].

**Fig 1:**
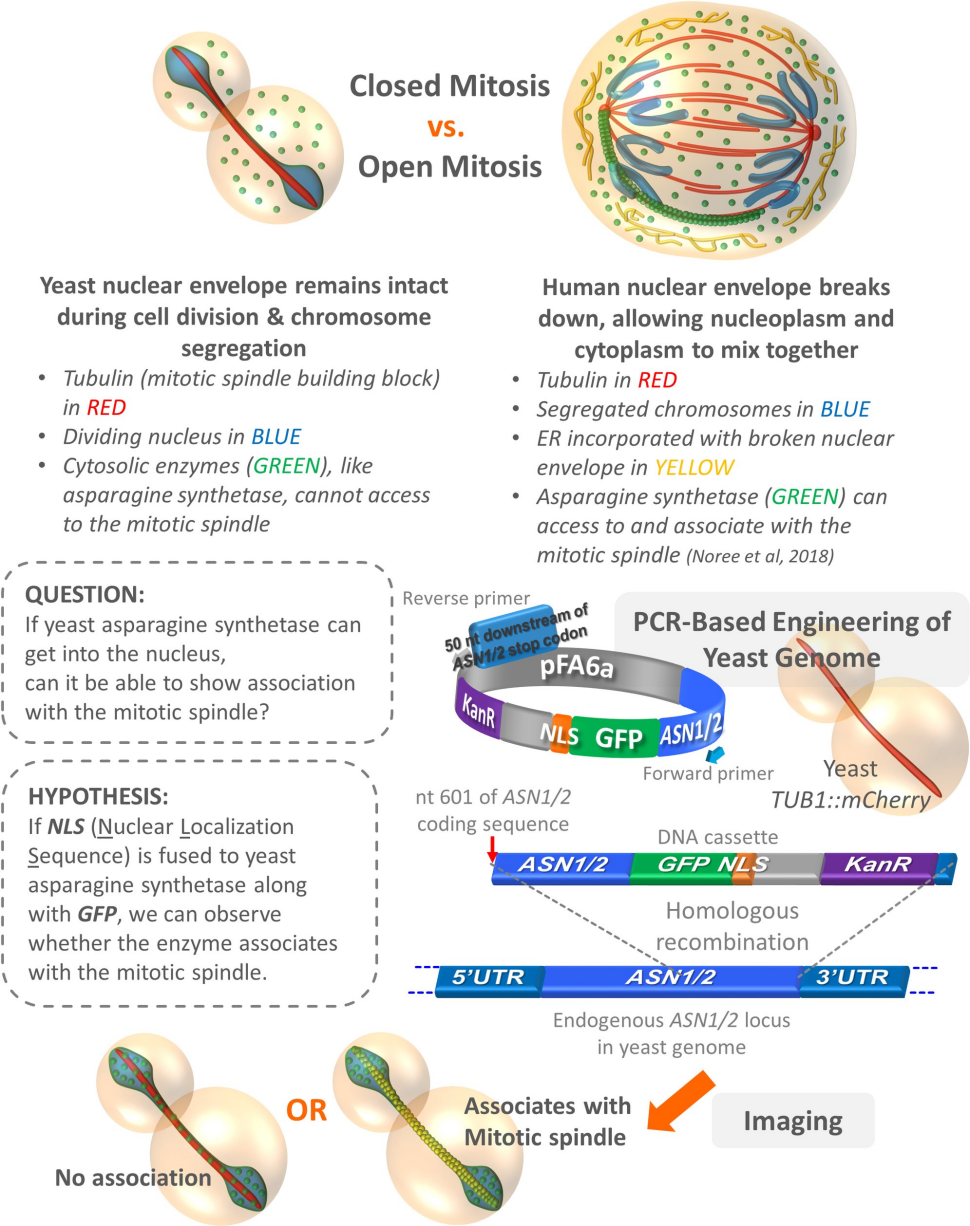
Observations, question, hypothesis, and experimental design. The differences between closed vs. open mitosis found in yeast and human cells, respectively, are described in the upper panel. An example showing human asparagine synthetase (green) lining up with the bottom left mitotic spindle (red) is illustrated (actually, the enzyme typically clusters around the entire mitotic spindles). The question raised in this study, hypothesis to be tested, and experimental design are shown in the lower panel.

Despite conservation of critical amino acid residues and structural domains shared between yeast and human asparagine synthetases [17], the different locations of yeast and human asparagine synthetase clusters (cytoplasmic filament vs. mitotic spindle-associated) have led to the question if yeast asparagine synthetases can also co-localize with the mitotic spindle. However, it is known that cell division in yeast differs from in human cells because yeast nuclear envelope never breaks down (known as closed mitosis) [29]. As a result, yeast asparagine synthetase cannot get into the nucleus by itself (Fig 1). In this study, we engineered the yeast genome such that yeast asparagine synthetases (both wild-type and loss-of-function mutant versions) were expressed as GFP and NLS fusion proteins, allowing translocation of the enzymes into the nucleus to microscopically observe whether they can associate with the mitotic spindle. The findings from our artificial yeast constructs have provided further supporting evidence that the association of yeast Asn1/2p with the mitotic spindle is possible, as previously observed for their human homolog, if they can get into the nucleus.

## Results and discussion

To help yeast asparagine synthetases move across the nuclear membrane, we decided to introduce the coding sequence of green fluorescent protein (*GFP*; to visualize the localization of the fluorescently tagged protein under fluorescence microscope) and also the coding sequence of nuclear localization signal (*NLS*; to make the fusion protein capable of nuclear transport) [30] downstream of the coding sequence of *ASN1* (or *ASN2*), in the yeast genome (Fig 1). This approach can ensure that the expression of GFP-NLS-tagged asparagine synthetases (Asn1p-GFP-NLS and Asn2p-GFP-NLS) is still under the control of their endogenous promoter and each of them is made from only a single copy of gene per cell, thus ruling out the overexpression issue which might cause the assembly artifact [31].

**Fig 2:**
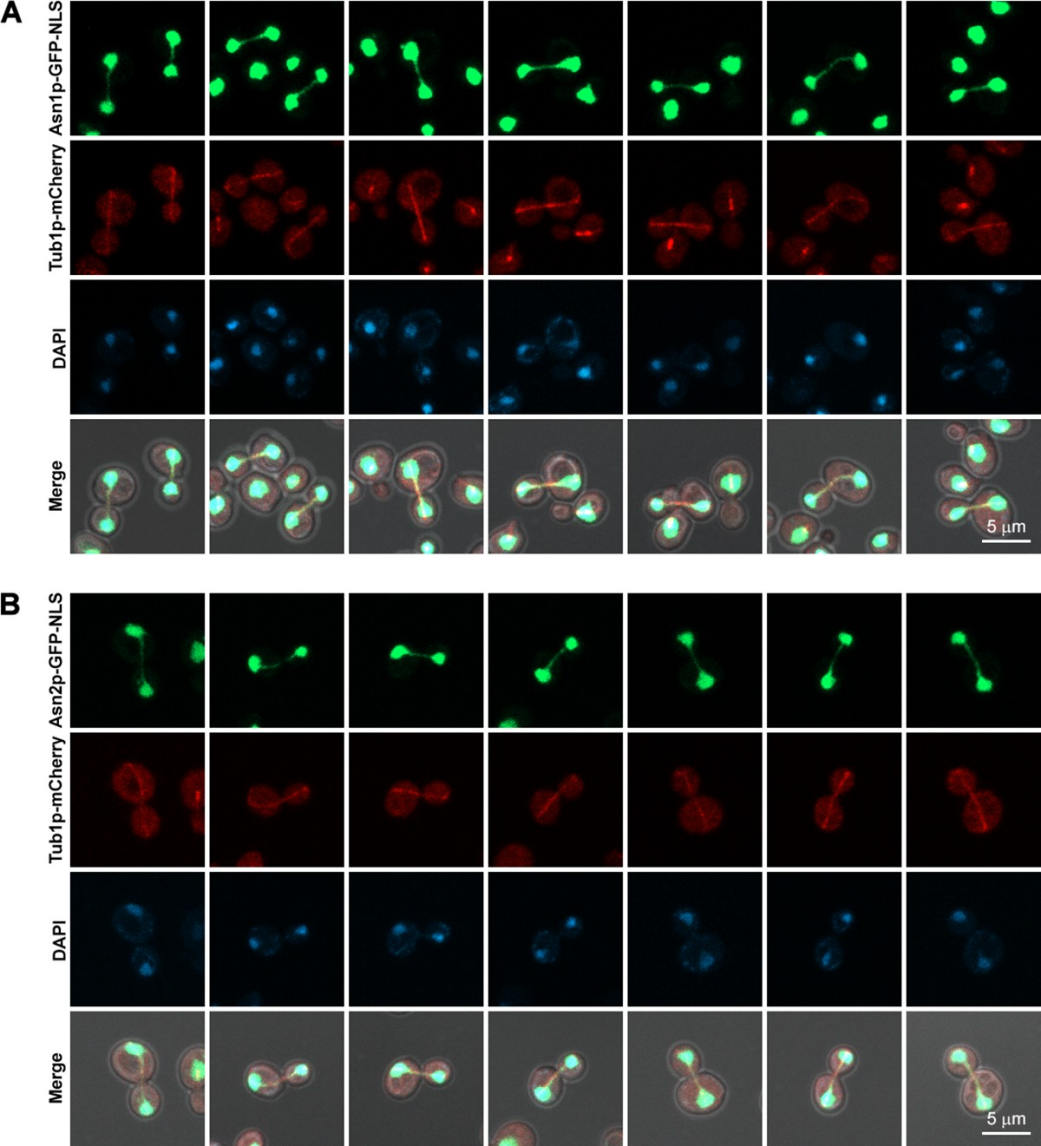
Asn1p-GFP-NLS and Asn2p-GFP-NLS both showed co-localization with the mitotic spindles (Tub1p-mCherry). (A) Yeast *ASN1*::*GFP*::*NLS TUB1*::*mCherry* and (B) *ASN2*::*GFP*::*NLS TUB1*::*mCherry* were cultured in YPD at 30°C with shaking to the log-phase, fixed with formaldehyde [10:1 (v/v) culture to 37% formaldehyde], and stained with DAPI (2 μg/ml) before imaging. Representative images of fixed cells were captured in Z-stack for 1–3 μm, and compressed into 2D images using maximum projection. At least 100 actively dividing cells of each strain were inspected.

To check the co-localization, *TUB1* (coding for alpha-tubulin, a building block of mitotic spindle), was also chromosomally engineered to have the coding sequence of *mCherry* (red fluorescent protein) downstream of *TUB1* coding sequence, therefore producing Tub1p-mCherry. The two genomically engineered yeast strains can successfully express Asn1p-GFP-NLS with Tub1p-mCherry, and Asn2p-GFP-NLS with Tub1p-mCherry, respectively. And, the GFP-NLS-tagged asparagine synthetases showed positive fluorescent signal in the nucleus (the cytoplasm is a predominant or residential location of typical asparagine synthetases), indicating that the GFP-NLS introduced to the C-terminus of the enzyme was functional. Both yeast strains showed a robust co-localization of GFP-NLS-tagged asparagine synthetase and mCherry-tagged alpha-tubulin in the dividing nuclei as shown in Fig 2 (Representative 3D images shown in S1 Fig).

**Fig 3:**
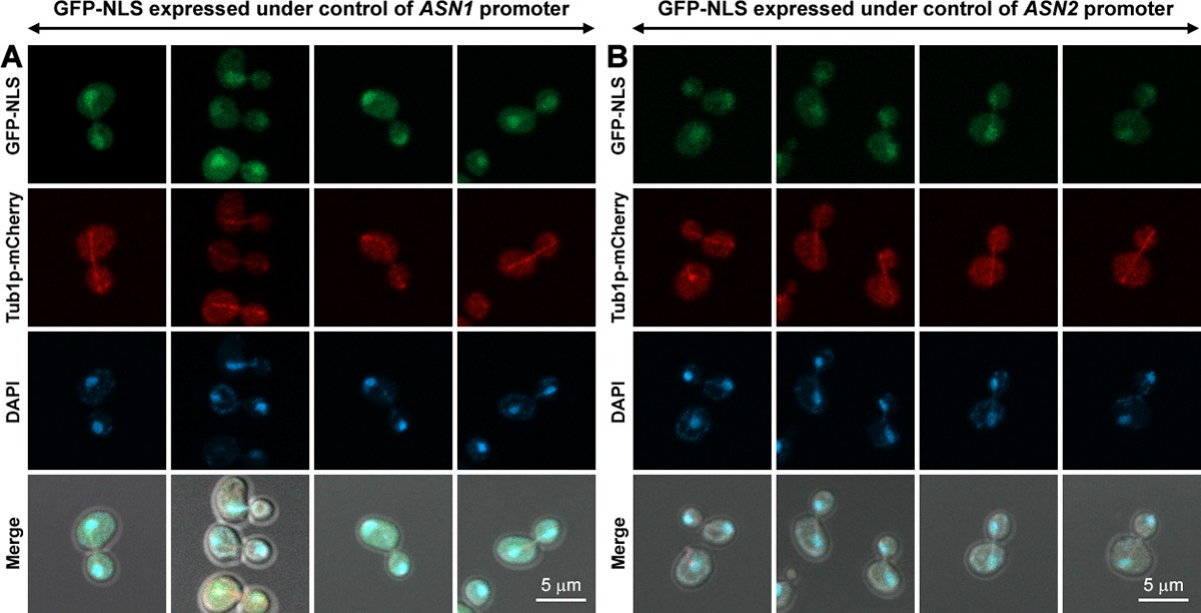
GPF-NLS fusion protein chromosomally expressed under either *ASN1* or *ASN2* promoter was used as a control for co-localization study of Asn1p-GFP-NLS (or Asn2p-GFP-NLS) and Tub1p-mCherry. (A) Yeast *P*_*ASN1*_::*GFP*::*NLS TUB1*::*mCherry* and (B) *P*_*ASN2*_::*GFP*::*NLS TUB1*::*mCherry* were cultured, in YPD at 30°C with shaking to the log-phase, fixed with formaldehyde [10:1 (v/v) culture to 37% formaldehyde], and stained with DAPI (2 μg/ml) before imaging. Representative images of fixed cells were captured in Z-stack for 1–3 μm, and compressed into 2D images using maximum projection. At least 100 actively dividing cells of yeast *P*_*ASN1*_::*GFP*::*NLS TUB1*::*mCherry* were inspected. For yeast *P*_*ASN2*_::*GFP*::*NLS TUB1*::*mCherry*, 62 actively dividing cells were inspected.

However, before making a conclusion that the mitotic spindle association feature is actually (but hiddenly) conserved in yeast asparagine synthetases (as being obscured by closed mitosis), the yeast genome was modified, similar to what we mentioned above, except that the whole coding sequence of *ASN1* (or *ASN2*) was removed from its chromosomal locus and replaced with *GFP* and *NLS*, instead. Thus, the GFP-NLS protein was genomically expressed under *ASN1* (or *ASN2*) promoter (*P*_*ASN1*_ / *P*_*ASN2*_) and can be used as a control to confirm if the co-localization observed between Asn1p-GFP-NLS (or Asn2p-GFP-NLS) and Tub1p-mCherry is real. In Fig 3, the fluorescent signal of GFP-NLS can be detected in both cytoplasm and nucleus, but much stronger in the nucleus. Since these two control yeast strains also showed GFP signals in the cytoplasm, even though being fused with NLS, we therefore measured the fluorescence intensity in the cytoplasm and in the nucleus of each cell. The ratio of fluorescence intensity in the nucleus to fluorescence intensity in the cytoplasm was about 3.1 for GFP-NLS expressed under *P*_*ASN1*_ (n = 100) and 2.7 for GFP-NLS expressed under *P*_*ASN2*_ (n = 62) (S1 and S2 Files). However, the lineup of GFP-NLS with the mitotic spindle could not be clearly detected when compared to those of Asn1p-GFP-NLS and Asn2p-GFP-NLS (noting that the imaging settings were the same for all yeast constructs).

Furthermore, Although the protein levels of Asn2p-GFP-NLS and Asn2p(R343A)-GFP-NLS were higher than that of their GFP-NLS control (expressed under *P*_*ASN2*_) (2.80 and 2.45 fold average of two independent experiments, respectively), but the protein levels of Asn1p-GFP-NLS and Asn1p(R344A)-GFP-NLS were 0.76 and 0.54 fold (average of two independent experiments), respectively, relative to that of the GFP-NLS control (expressed under *P*_*ASN1*_), arguing that the lineup of nuclear targeted Asn1/2p with the mitotic spindle is not influenced by the expression level (S2 Fig and S3 File).

**Fig 4:**
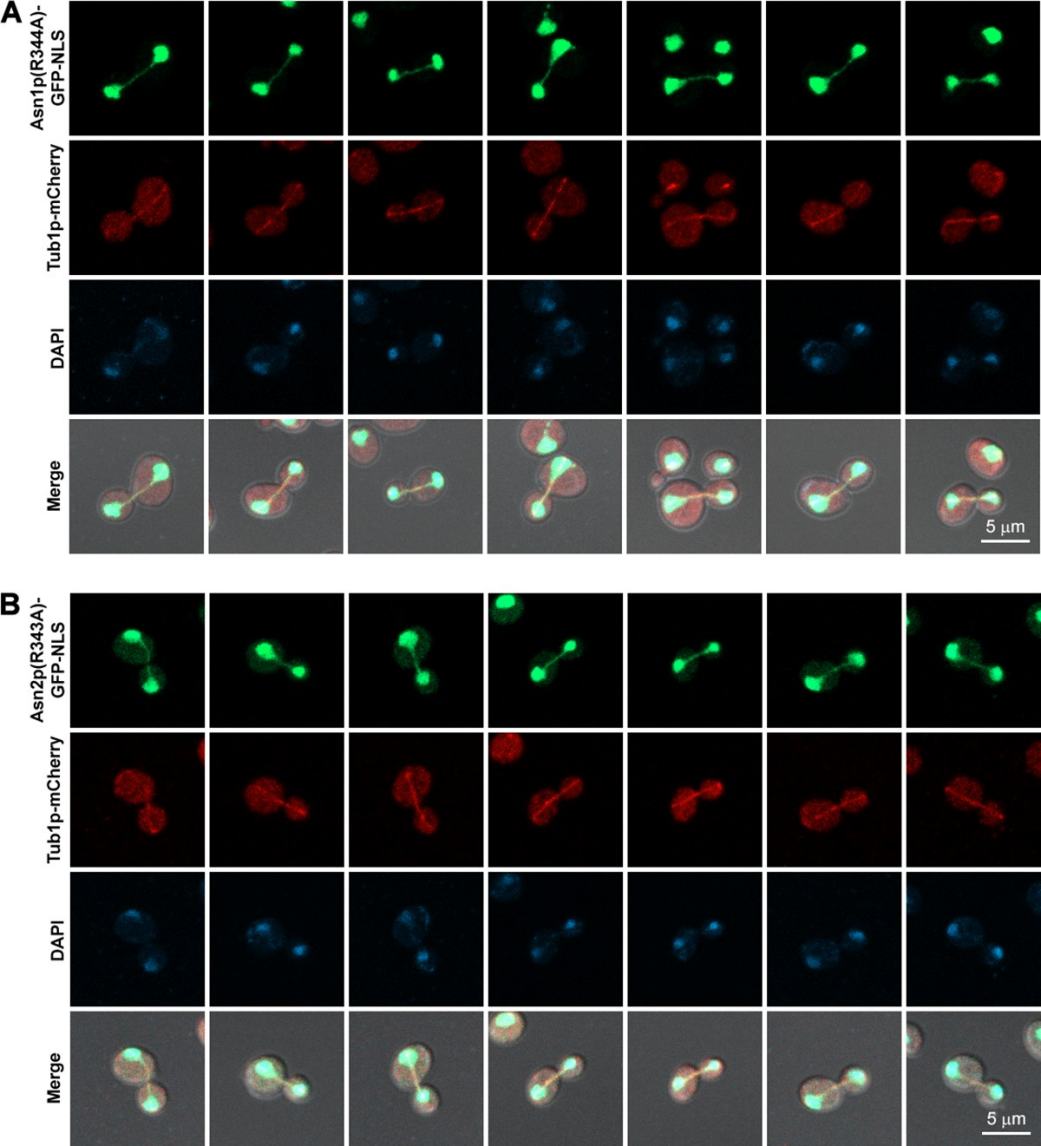
Activity-disrupted asparagine synthetases Asn1p(R344A)-GFP-NLS and Asn2p(R343A)-GFP-NLS were still able to co-localize with the mitotic spindles (Tub1p-mCherry). (A) Yeast *asn1(R344A)*::*GFP*::*NLS TUB1*::*mCherry* and (B) *asn2(R343A)*::*GFP*::*NLS TUB1*::*mCherry* were cultured in YPD at 30°C with shaking to the log-phase, fixed with formaldehyde [10:1 (v/v) culture to 37% formaldehyde], and stained with DAPI (2 μg/ml) before imaging. Representative images of fixed cells were captured in Z-stack for 1–3 μm, and compressed into 2D images using maximum projection. At least 100 actively dividing cells of each strain were inspected.

Since a typical role of any metabolic enzyme is to catalyze certain biochemical reaction(s) in living cells, we asked if the association of asparagine synthetase with the mitotic spindle would rely on its enzymatic activity. So, another two yeast strains were constructed genomically expressing loss-of-activity mutant enzyme [17, 32–34] Asn1p(R344A)-GFP-NLS along with Tub1p-mCherry, and Asn2p(R343A)-GFP-NLS with Tub1p-mCherry, respectively (for the amino acid sequence alignment showing the conserved arginine residue R340 in hASNS, R344 in yeast Asn1p, and R343 in yeast Asn2p, see S3 Fig). Even though the activity of mutant Asn1/2p was disrupted, their co-localization with the mitotic spindle can still be observed (Fig 4) (Representative 3D images shown in S1 Fig). This indicates that asparagine synthetase associates with the mitotic spindle in an enzymatic activity-independent manner, thus implying that, in higher eukaryotic cells, its moonlighting role in cell division might occur without the coordination with its biochemical role.

**Fig 5:**
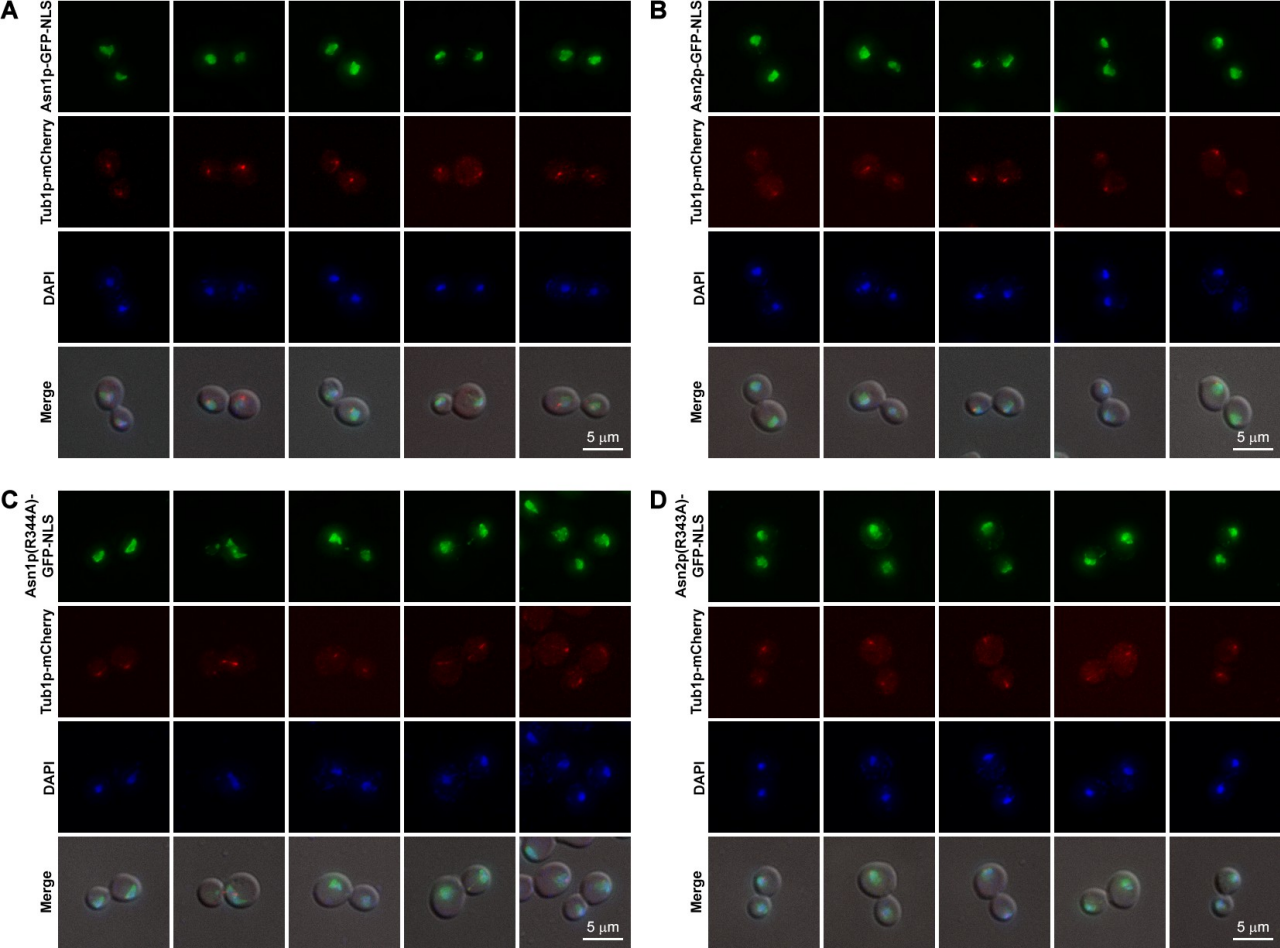
Association between nuclear targeted functional and loss-of-function Asn1/2p and mitotic spindle disappeared after nocodazole treatment. (A) Yeast *ASN1*::*GFP*::*NLS TUB1*::*mCherry*, (B) *ASN2*::*GFP*::*NLS TUB1*::*mCherry*, (C) *asn1(R344A)*::*GFP*::*NLS TUB1*::*mCherry*, and (D) *asn2(R343A)*::*GFP*::*NLS TUB1*::*mCherry* were cultured in YPD containing 200 ng/ml nocodazole at 30°C with shaking to the log-phase (4 hours), fixed with formaldehyde [10:1 (v/v) culture to 37% formaldehyde], and stained with DAPI (2 μg/ml) before imaging. Representative images of nocodazole-treated cells were captured in Z-stack for 1–3 μm, and compressed into 2D images using maximum projection. At least 100 cells of each strain were inspected.

Next, we asked if the disruption of mitotic spindle formation by a microtubule/mitotic spindle destabilizing agent, nocodazole, could prevent the association between nuclear targeted yeast Asn1/2p and the mitotic spindle. In agreement with our previous study with human cell lines [28], the diluted yeast cultures treated with 200 ng/ml nocodazole for 4 hours did not show any association of Asn1p-GFP-NLS, Asn2p-GFP-NLS, Asn1p(R344A)-GFP-NLS, or Asn2p(R343A)-GFP-NLS with the de-polymerized mitotic spindle (Fig 5). However, we noticed that under this treatment condition, there were still some cells (<5%) with intact mitotic spindle (not shown here), but we didn’t want to use higher concentrations of nocodazole or extend the treatment incubation time which could trigger cell death.

Altogether, our findings suggest that yeast asparagine synthetases can feature the mitotic spindle association once they are re-localized to the nucleus. Anyway, further experiments should be conducted to reveal how the asparagine synthetase of higher eukaryotic cells (with open mitosis) takes part in the chromosome segregation and cell division. In case of yeast, perhaps, a subpopulation of yeast asparagine synthetases might be translocated across the nuclear membrane to do the same thing, but we just never take a close look at it, yet.

The concept of multi-function (metabolic and non-metabolic), multi-organization (freely soluble, oligomerized, and higher-order), and multi-location (being temporally in different subcellular compartments) of metabolic enzymes is currently getting more attention [35–37]. And, it is very interesting when they come with a variety of utilizations and regulations in different species. Asparagine synthetase is one of them and it can potentially be used as a model enzyme for further studies to gain more insights into its complicated and unprecedented functions that are influenced by intrinsic factors, micro-, and macro-environments.

## Conclusions

Yeast asparagine synthetases Asn1p and Asn2p were re-localized to nucleus in order to investigate if they could line up with the mitotic spindle during yeast cell division. The co-localization data from both functional and activity-disrupted Asn1/2p suggest that asparagine synthetase associates with the mitotic spindle in an activity-independent manner, implying that the enzyme, in higher eukaryotes with open mitosis, might have a moonlighting function in cell division, in addition to its catalytic function.

## Materials and methods

### Bacteria, yeast, growth and selection media

*Escherichia coli* DH5α was used as bacterial host for transformation and propagation of the recombinant plasmids. LB medium [0.5% (w/v) yeast extract (BD Biosciences), 1% (w/v) Bacto^TM^ Tryptone (BD Biosciences), 1% (w/v) sodium chloride (BDH Prolabo)] supplemented with 100 μg/ml ampicillin (PanReac AppliChem) was used for selection. Bacterial cultures were maintained at 37°C.

Yeast *BY4741* (*MATa his3Δ1 leu2Δ0 met15Δ0 ura3Δ0*), used as a background strain for yeast chromosomal gene modifications, was purchased from Thermo Fisher Scientific. YPD [1% (w/v) yeast extract, 2% (w/v) Bacto^TM^ Peptone (BD Biosciences), and 2% (w/v) dextrose (Sigma-Aldrich)] medium was used for general cultures. G418 (PanReac AppliChem) (400 μg/ml final concentration) and Hygromycin B (Merck) (200 μg/ml final concentration) were used for selecting yeast transformants as indicated. All yeast strains were maintained at 30°C.

### PCR-based site-directed mutagenesis for creating plasmids pFA6a-ASN1-GFP-NLS-kanMX6 and pFA6a-ASN2-GFP-NLS-kanMX6

pFA6a-ASN1-GFP-kanMX6 [17] and pFA6a-ASN2-GFP-kanMX6 (constructed and verified using the same protocols used for creating pFA6a-ASN1-GFP-kanMX6; *ASN2* coding sequence was amplified from genomic DNA of yeast BY4741 and cloned into *Sal*I and *Sma*I recognition sites of pFA6a-GFP-kanMX6; primers used for the recombinant plasmid cloning and verification by DNA sequencing are shown in S1 Table) were used as DNA templates to introduce *NLS* (5’- CCA CCA AAA AAA AAA AGA AAA GTT -3’, coding for PPKKKRKV) [30] to be downstream of *GFP* coding sequence within the plasmids. All primers used for mutagenization are listed in S1 Table. Primers were first phosphorylated at their 5’ ends with T4 Polynucleotide Kinase (New England Biolabs). PCR-based site-directed mutagenesis reactions were set up using KOD Hot Start DNA Polymerase kit (Merck). The plasmid templates were removed by *Dpn*I (New England Biolabs) treatment. The mutagenized PCR products were purified using GenepHlow^TM^ Gel/PCR Kit (Geneaid). Ligations were set up using T4 DNA Ligase (New England Biolabs) to make mutagenized PCR products (linear plasmids) become circular prior to bacterial transformation. After transformation using heat shock method, plasmids were propagated and isolated from the selected transformants using Presto^TM^ Mini Plasmid Kit (Geneaid), and were then verified by DNA sequencing (Macrogen, South Korea). The resulting plasmids after mutagenization were referred to as pFA6a-ASN1-GFP-NLS-kanMX6 and pFA6a-ASN2-GFP-NLS-kanMX6, respectively.

### PCR-based site-directed mutagenesis for creating plasmids pFA6a-asn1(R344A)-GFP-NLS-kanMX6 and pFA6a-asn2(R343A)-GFP-NLS-kanMX6

pFA6a-ASN1-GFP-NLS-kanMX6 and pFA6a-ASN2-GFP-NLS-kanMX6 were used as DNA templates to introduce R344A (for *ASN1*) or R343A mutation (for *ASN2*). All primers used for mutagenization are listed in S1 Table. PCR-based site-directed mutagenesis was performed using the protocols as mentioned above. The resulting plasmids after mutagenization and confirmation by DNA sequencing were referred to as pFA6a-asn1(R344A)-GFP-NLS-kanMX6 and pFA6a-asn2(R343A)-GFP-NLS-kanMX6, respectively.

### Construction of double-fluorescently tagged yeast strains for co-localization assays

First, yeast *TUB1*::*mCherry* was created using yeast *BY4741* as a base strain. pBS35 (Addgene, a gift from J. Wilhelm, UC San Diego) was used as a DNA template for making DNA cassette harboring (sequence in order from 5’ to 3’): 50 bp upstream of the *TUB1* stop codon, *mCherry* coding sequence, Hygromycin resistance gene, and 50 bp downstream of the *TUB1* stop codon. The PCR reaction was set up using the KOD Hot Start DNA Polymerase kit. Yeast *BY4741* was transformed with the purified DNA cassette using lithium acetate/polyethylene glycol transformation method. YPD medium supplemented with Hygromycin B was used for selection. PCR was employed to verify that *mCherry* coding sequence had been successfully fused to *TUB1* in the yeast genome. The primers used for mCherry fusion are shown in S1 Table.

After getting yeast *TUB1*::*mCherry*, four recombinant plasmids pFA6a-ASN1-GFP-NLS-kanMX6, pFA6a-ASN2-GFP-NLS-kanMX6, pFA6a-asn1(R344A)-GFP-NLS-kanMX6, and pFA6a-asn2(R343A)-GFP-NLS-kanMX6 were used as DNA templates for making four different DNA cassettes harboring (sequence in order from 5’ to 3’): *ASN1* (or *ASN2*) coding sequence (nt601 onward) with or without R344A (for *ASN1*) or R343A (for *ASN2*) mutation, *GFP*, *NLS*, Kanamycin resistance gene, and 50 bp downstream of the *ASN1* (or *ASN2*) stop codon. The PCR reactions were set up using the KOD Hot Start DNA Polymerase kit. Yeast *TUB1*::*mCherry* was transformed with each purified DNA cassette using lithium acetate/polyethylene glycol transformation method. YPD medium supplemented with G418 and Hygromycin B was used for selection. The positive yeast transformants were initially screened under the fluorescence microscope by checking the localization patterns of GFP-tagged asparagine synthetase (nuclear localization if successfully tagged with NLS vs. cytoplasmic localization if negative), and were then confirmed by sending out the PCR products of the genomic DNA isolated from the yeast constructs (to be verified) for DNA sequencing. The resulting strains were referred to as: (1) *ASN1*::*GFP*::*NLS TUB1*::*mCherry*, (2) *ASN2*::*GFP*::*NLS TUB1*::*mCherry*, (3) *asn1(R344A)*::*GFP*::*NLS TUB1*::*mCherry*, and (4) *asn2(R343A)*::*GFP*::*NLS TUB1*::*mCherry*, respectively.

For the control yeast strains (*P*_*ASN1(or ASN2)*_::*GFP*::*NLS TUB1*::*mCherry*), PCR was also used to prepare the DNA cassettes harboring (sequence in order from 5’ to 3’): 50 bp upstream of the *ASN1* (or *ASN2*) start codon, start codon with *GFP*, *NLS*, Kanamycin resistance gene, and 50 bp downstream of the *ASN1* (or *ASN2*) stop codon. Yeast *TUB1*::*mCherry* was transformed with each purified DNA cassette, and the transformants were selected and finally verified using the same protocols, as described above.

After genomic sequence verification, at least 3 different clones were collected and inspected for each yeast construct to make sure that they all exhibited the same phenotype.

The primers for making the DNA cassettes for yeast transformations, preparing PCR products (of genomic DNA isolated from yeast transformants) for strain verification by DNA sequencing, and the sequencing primers are shown in S1 Table.

### Cell imaging

An aliquot of the overnight culture of each yeast strain (1:30–1:50 diluted) was transferred to a new culture tube with fresh YPD. The diluted culture was grown to log phase at 30°C with shaking for 4 hours. Cells were fixed with formaldehyde by using 100 μl 37% formaldehyde per 1 ml of cell culture. DAPI (Sigma-Aldrich, 2 μg/ml final concentration) was also added to the tube during cell fixation. After fixing cells at room temperature for 15 min with shaking in the dark, the cells were washed twice with sterile water, and then resuspended in either 1xPBS or 1M sorbitol. Wet slides were prepared by dropping the fixed cell suspension (about 10 μl) onto a slide (Shandon SuperFrost Plus, Thermo Fisher Scientific), covering with a coverslip (Menzel Gläser, Thermo Fisher Scientific), blotting off excess liquid to prevent cells from floating around, and then sealing the edges of the coverslip with nail polish.

Images (in Figs 2–4) were taken in Z-stack (1–3 μm) using the Carl Zeiss LSM800 (with AiryScan) and Plan-Apochromat 63X/1.4 Oil DIC ∞/0.17 objective lens. Z-stack images were then compressed into a single image with maximum projection using Zen Blue software version 2.1.57.1000. Images (in Fig 5) were taken in Z-stack (1–3 μm) using the DeltaVision Ultra with Olympus PlanApo N 60X/1.42 Oil objective lens. Z-stack images were deconvoluted and compressed into a single image with maximum projection using softWoRx software version 7.2.1 (the Advanced Cell Imaging Center, Institute of Molecular Biosciences, Mahidol University).

The fluorescence intensity within the nucleus and the cytoplasm of yeast *P*_*ASN1*_::*GFP*::*NLS TUB1*::*mCherry* and yeast *P*_*ASN2*_::*GFP*::*NLS TUB1*::*mCherry* was measured using Zen Blue software. The ratio of fluorescence intensity in the nucleus to fluorescence intensity in the cytoplasm was calculated for each cell, and the average value for each strain was then reported.

### Nocodazole treatment

An aliquot of the overnight culture of each yeast strain (1:30–1:50 diluted) was transferred to a new culture tube with fresh YPD containing 200 ng/ml nocodazole (Sigma-Aldrich). The diluted culture was grown at 30°C with shaking for 4 hours before fixing the cells with formaldehyde and preparing slides for imaging, using the same protocols as mentioned above.

### Western blot analysis

All yeast strains were cultured in YPD to log-phase at 30°C with shaking. One OD_600_ cells were taken from each culture for preparing whole cell extract. After centrifugation at 6,000 rpm for 2 min and removal of supernatant, cells were lysed in 100 μl SDS-PAGE sample loading buffer containing 4M Urea (Research Organics), (1:20) beta-mercaptoethanol (PanReac Applichem) and (1:1,000) protease inhibitor cocktail (Sigma-Aldrich). About 50 μl glass beads (425–600 μm) (Sigma-Aldrich) were added to help in cell lysis during the vigorous vortex for 1 min. After boiling protein samples at 95°C for 10 min and placing on ice for 5 min, they were centrifuged at 10,000 rpm for 1 min at room temperature. The total protein samples were resolved on 8% SDS-PAGE [15 μl loaded for detection with anti-GFP, and 5 μl loaded for detection with anti-phosphoglycerate kinase (anti-Pgk1p)]. AccuProtein Chroma prestained protein marker (Enzmart Biotech) was used to identify the approximate size of Asn1p-GFP-NLS (92.71 kDa), Asn1p(R344A)-GFP-NLS (92.62 kDa), GFP-NLS (expressed under *P*_*ASN1*_) (27.89 kDa), Asn2p-GFP-NLS (92.83 kDa), Asn2p(R343A)-GFP-NLS (92.74 kDa), GFP-NLS (expressed under *P*_*ASN2*_) (27.89 kDa), and Pgk1p (44.74 kDa, as an internal loading control). The resolved proteins were transferred to PVDF membrane (Bio-Rad) using Trans-Blot® SD Semi-Dry Transfer Cell (Bio-Rad). Western blotting was performed using standard protocol. Rabbit anti-GFP polyclonal antibody (A01388; GenScript; 1:5,000) was used for detecting Asn1p-GFP-NLS, Asn1p(R344A)-GFP-NLS, GFP-NLS (expressed under *P*_*ASN1*_), Asn2p-GFP-NLS, Asn2p(R343A)-GFP-NLS, and GFP-NLS (expressed under *P*_*ASN2*_). Mouse anti-Pgk1p (22C5D8; Thermo Fisher Scientific; 1:5,000) was used for detecting internal loading control. The secondary antibodies for detection were HRP-conjugated goat anti-rabbit IgG (Sigma-Aldrich; 1:5,000) and HRP-conjugated goat anti-mouse IgG (Sigma-Aldrich; 1:5,000), respectively. ECL Western Blotting Detection System (GE Healthcare) was used to develop the chemiluminescent signals before exposure to X-ray films (Fujifilm). Two independent experiments were performed to confirm the results. ImageJ (W. Rasband, National Institute of Health, USA) was used to quantify the blots. The expression ratio of GFP: Pgk1p of each sample was calculated, and then the fold-change relative to the control was presented.

## Supporting information

S1 Fig Representative 3D images of yeast *ASN1*::*GFP*::*NLS TUB1*::*mCherry*, *ASN2*::*GFP*::*NLS TUB1*::*mCherry*, *asn1(R344A)*::*GFP*::*NLS TUB1*::*mCherry*, and *asn2(R343A)*::*GFP*::*NLS TUB1*::*mCherry*, respectively. (TIF)

S2 Fig Western blot analysis to check the protein levels of Asn1p-GFP-NLS and Asn1p(R344A)-GFP-NLS compared to that of GFP-NLS (expressed under *P*_*ASN1*_), and the protein levels of Asn2p-GFP-NLS and Asn2p(R343A)-GFP-NLS compared to that of GFP-NLS (expressed under *P*_*ASN2*_). All yeast strains were grown to log-phase in YPD at 30°C with shaking. SDS-PAGE and Western blotting was performed to detect the GFP fusion proteins with anti-GFP and Pgk1p (as internal loading control) with anti-Pgk1p, respectively, in different blots. ImageJ was used to quantify the blots. The degraded bands of GFP-tagged asparagine synthetases were also included in the quantitation to prevent any bias. The GFP: Pgk1p normalized data of each sample was calculated, then the fold-change relative to the GFP-NLS control was presented. Two independent experiments (A) and (B) were performed.
(TIF)

S3 Fig Amino acid sequence alignment of human and yeast asparagine synthetases. Amino acid sequences of hASNS (P08243), yeast Asn1p (P49089), and yeast Asn2p (P49090) were aligned using CLUSTALO available at www.uniprot.org. They show 200 identical and 150 similar amino acid residues. The red box indicates the conserved arginine residue, critical for synthetase activity, located at position 340 of hASNS, 344 of yeast Asn1p, and 343 of yeast Asn2p, respectively (the positions of amino acid residues mentioned in this study are based on the protein sequences still having a beginning methionine).
(TIF)

S1 Table List of primers (synthesized by ValueGene, USA, and Macrogen, South Korea) used for recombinant plasmid cloning and modifications (by PCR-based site-directed mutagenesis), making DNA cassettes for yeast transformations, and preparing PCR products (of isolated yeast genomic DNA) for strain verification by DNA sequencing. (PDF)

S1 File The data of fluorescence intensity within the nucleus and the cytoplasm of yeast *P*_*ASN1*_::*GFP*::*NLS TUB1*::*mCherry*. (PDF)

S2 File The data of fluorescence intensity within the nucleus and the cytoplasm of yeast *P*_*ASN2*_::*GFP*::*NLS TUB1*::*mCherry*. (PDF)

S3 File The data of Western blot quantification for S2 Fig. (PDF)
