# Supplementary figures and images for: Nuclear targeted Saccharomyces cerevisiae asparagine synthetases associate with the mitotic spindle regardless of their enzymatic activity
Source: PLoS One. 2020 Dec 21;15(12):e0243742. doi: 10.1371/journal.pone.0243742 (PMC7751962; doi:10.1371/journal.pone.0243742)

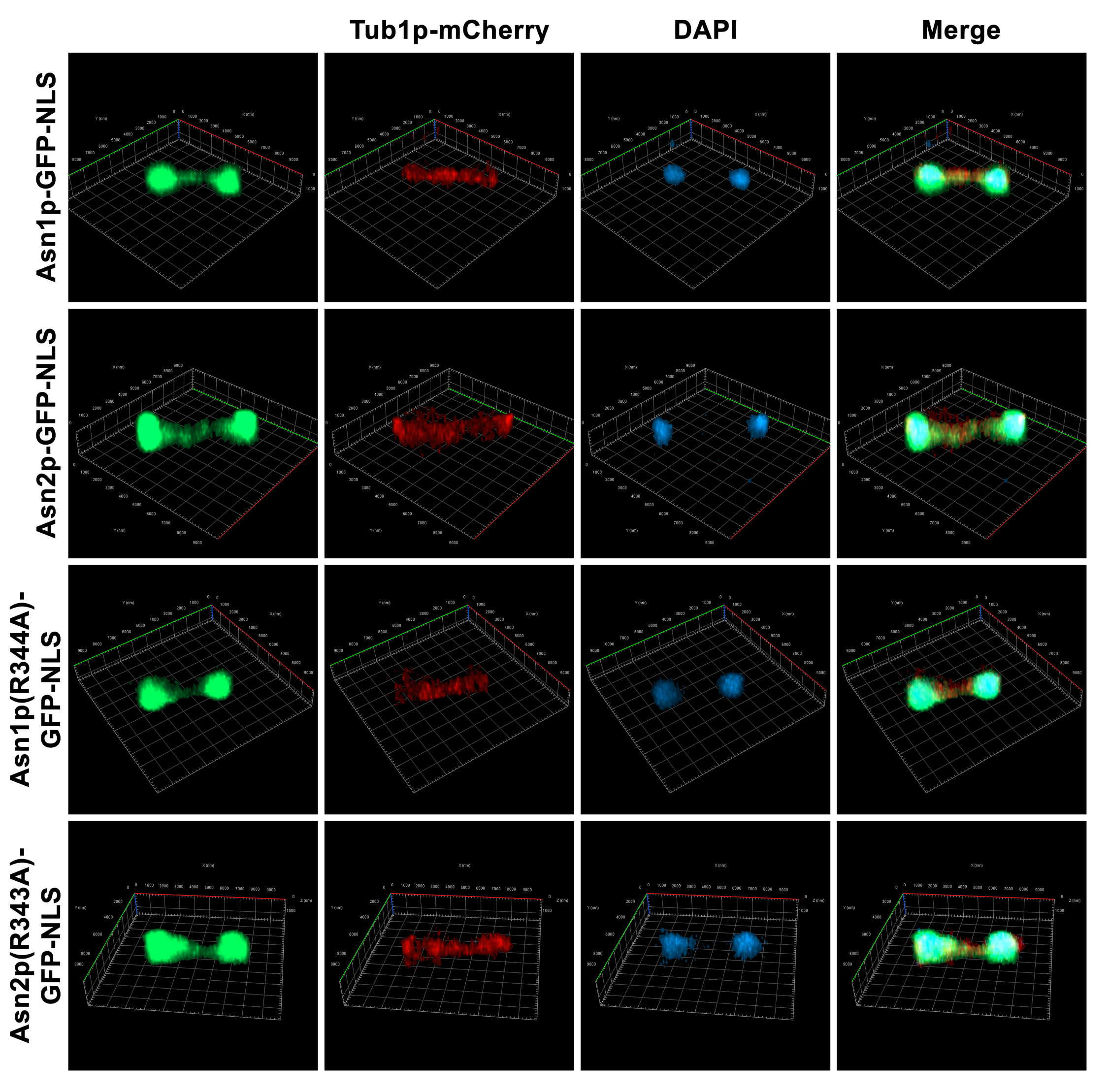

Supplement: S1 Fig — (TIF) [file pone.0243742.s001.tif]

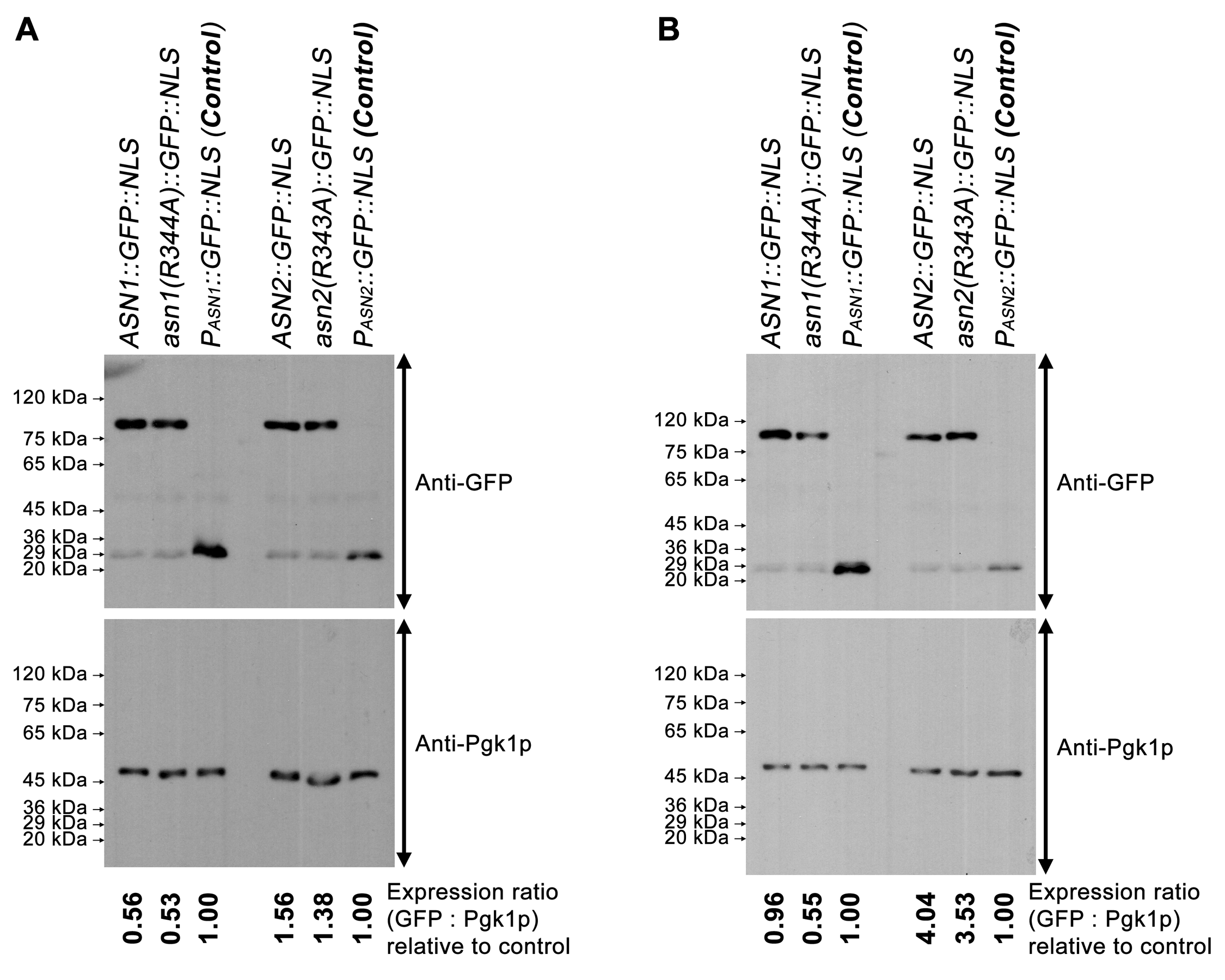

Supplement: S2 Fig — All yeast strains were grown to log-phase in YPD at 30°C with shaking. SDS-PAGE and Western blotting was performed to detect the GFP fusion proteins with anti-GFP and Pgk1p (as internal loading control) with anti-Pgk1p, respectively, in different blots. ImageJ was used to quantify the blots. The degraded bands of GFP-tagged asparagine synthetases were also included in the quantitation to prevent any bias. The GFP: Pgk1p normalized data of each sample was calculated, then the fold-change relative to the GFP-NLS control was presented. Two independent experiments (A) and (B) were performed. (TIF) [file pone.0243742.s002.tif]

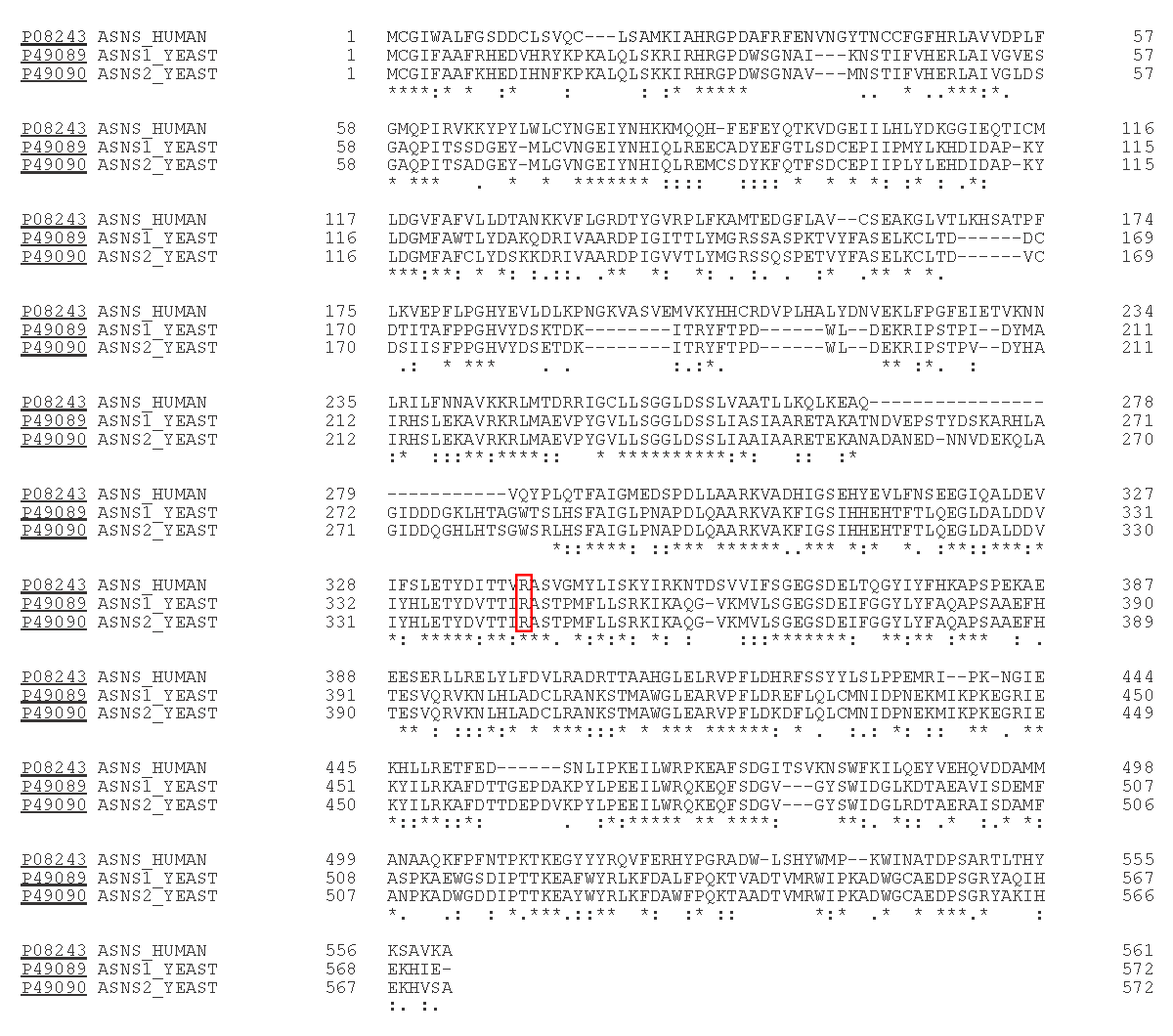

Supplement: S3 Fig — Amino acid sequences of hASNS (P08243), yeast Asn1p (P49089), and yeast Asn2p (P49090) were aligned using CLUSTALO available at www.uniprot.org. They show 200 identical and 150 similar amino acid residues. The red box indicates the conserved arginine residue, critical for synthetase activity, located at position 340 of hASNS, 344 of yeast Asn1p, and 343 of yeast Asn2p, respectively (the positions of amino acid residues mentioned in this study are based on the protein sequences still having a beginning methionine). (TIF) [file pone.0243742.s003.tif]
