# Supplementary material for: Nuclear targeted Saccharomyces cerevisiae asparagine synthetases associate with the mitotic spindle regardless of their enzymatic activity
Source: PLoS One. 2020 Dec 21;15(12):e0243742. doi: 10.1371/journal.pone.0243742 (PMC7751962; doi:10.1371/journal.pone.0243742)
Supplement: S1 Table — (PDF) [file pone.0243742.s004.pdf]

S1 Table. List of primers (synthesized by ValueGene, USA, and Macrogen, South Korea) used for recombinant plasmid cloning and modifications (by PCR-based site-directed mutagenesis), making DNA cassettes for yeast transformations, and preparing PCR products (of isolated yeast genomic DNA) for strain verification by DNA sequencing.

Primer	Sequence	Description	Used with	Product size
**Primers for making DNA cassette *TUB1::mCherry; hygR* (for transforming yeast *BY4741*)**<br>**Plasmid template:** pBS35<br>**Resulting yeast strain:** *TUB1::mCherry*				
CN0039	5’-GAGATTACATCGAAGTGGGTGCCGACTCATACGCTGAGGAAGAGGAATTTGGTCGACGGATCCCCGGG-3’	Forward; with 50 nt upstream of ***TUB1*** stop codon and homologous sequence to pBS35 (underlined)	CN0040	2,526 bp
CN0040	5’-ATATACTTGAAATATAGAAAGGATAAGGAGGTTGGGGGCGAGAGTGAACCATCGATGAATTCGAGCTCG -3’	Reverse; with 50 nt downstream of ***TUB1*** stop codon and homologous sequence to pBS35 (underlined)	CN0039	
**Primers for yeast genomic DNA verification by PCR**<br>**DNA template:** genomic DNA isolated from yeast *BY4741* transformed with *TUB1::mCherry; hygR* DNA cassette<br>**Verified strain:** *TUB1::mCherry*				
CN0046	5’- GCCAAACGTGCTTTCGTCCAC -3’	Forward; located at nt1201-1221 of ***TUB1*** coding sequence	JW1775	879 bp
JW1775	5'- CTACTTGTACAGCTCGTCCATGCC -3'	Reverse; located at nt688-711 of ***mCherry*** coding sequence	CN0046	
**Primers for cloning *ASN2* into pFA6a-GFP-kanMX6**<br>**DNA template:** genomic DNA isolated from yeast *BY4741*<br>**Resulting plasmid:** pFA6a-ASN2-GFP-kanMX6				
JW2024	5’-CTTGTCGACATGTGTGGTATCTTTGCAGCCTTC-3’	Forward, for cloning ***ASN2***, ***Sal*I** site underlined	JW2025	1,734 bp
JW2025	5’-CTTCCCGGGAGCACTGACGTGCTTTTCGTGTAT-3’	Reverse, for cloning ***ASN2***, ***Sma*I** site underlined	JW2024	
**Mutagenic primers for introducing *NLS* downstream of *GFP* coding sequence**<br>**Plasmid templates:**<br>(1) pFA6a-ASN1-GFP-kanMX6<br>(2) pFA6a-ASN2-GFP-kanMX6<br>**Resulting plasmids:**<br>(1) pFA6a-ASN1-GFP-NLS-kanMX6<br>(2) pFA6a-ASN2-GFP-NLS-kanMX6				
CN0054	5’-CCACCAAAAAAAAAAAGAAAAGTT**TAG**GGCGCGCCACTTCTAAATAAG -3’	Forward; *NLS* (underlined) + *GFP* stop codon (bold) and downstream sequence	CN0055	6,617 bp
CN0055	5’- TTTGTATAGTTCATCCATGCCATGTG -3’	Reverse; located upstream of *GFP* stop codon	CN0054	
**Primers for making DNA cassette *ASN1::GFP::NLS; kanR* (for transforming yeast *TUB1::mCherry*)**<br>**Plasmid template:** pFA6a-ASN1-GFP-NLS-kanMX6<br>**Resulting yeast strain:** *ASN1::GFP::NLS TUB1::mCherry*				
JW2038	5’- CGCATTCCTTCCACCCCAATAG -3’	Forward; located at nt601-622 of ***ASN1*** coding sequence	JW1993	3,614 bp
JW1993	5’-AAATATCTATAAGATTAATCCATAATTCTTTTTCTATTTTTTAATGTTATATCGATGAATTCGAGCTCG -3’	Reverse; with 50 nt downstream of ***ASN1*** stop codon and homologous sequence to	JW2038	

Primer	Sequence	Description	Used with	Product size
		plasmid template (underlined)		
**Primers for making DNA cassette P_{ASN1}*::GFP::NLS; kanR* (for transforming yeast *TUB1::mCherry*)**<br>**Plasmid template:** pFA6a-ASN1-GFP-NLS-kanMX6<br>**Resulting yeast strain:** P_{ASN1}*::GFP::NLS TUB1::mCherry*<br>[Verified by PCR (of genomic DNA isolated from the yeast transformants) with primers JW2010 and JW1623, producing 1,125 bp PCR product]				
CN0057	5' - AAAAGTATAACTTGCTTTACGCTAAGGATATAAATCGGACGTAACTTAAG**ATG**AGTAAAGGAGAAGAACTTTTCACTG -3'	Forward; having 50 nt upstream of ***ASN1*** start codon + ATG (bold) + ***GFP*** coding sequence (nt4-25)	JW1993	2,536 bp
JW1993	5'- AAATATCTATAAGATTAATCCATAATTCTTTTTCTATTTTTTAATGTTATATCGATGAATTCGAGCTCG -3'	Reverse; with 50 nt downstream of ***ASN1*** stop codon and homologous sequence to plasmid (underlined)	CN0057	
**Primers for making DNA cassette *ASN2::GFP::NLS; kanR* (for transforming yeast *TUB1::mCherry*)**<br>**Plasmid template:** pFA6a-ASN2-GFP-NLS-kanMX6<br>**Resulting yeast strain:** *ASN2::GFP::NLS TUB1::mCherry*				
JW2040	5'- CGTATCCCATCCACCCCAGTTGAC -3'	Forward; located at nt601-624 of ***ASN2*** coding sequence	JW1995	3,614 bp
JW1995	5'- CCGCATTTCTTGGTTCACTCGTCAATTATAAGAATACGATTGCGCTCGTA ATCGATGAATTCGAGCTCG -3'	Reverse; with 50 nt downstream of ***ASN2*** stop codon and homologous sequence to plasmid (underlined)	JW2040	
**Primers for making DNA cassette P_{ASN2}*::GFP::NLS; kanR* (for transforming yeast *TUB1::mCherry*)**<br>**Plasmid template:** pFA6a-ASN2-GFP-NLS-kanMX6<br>**Resulting yeast strain:** P_{ASN2}*::GFP::NLS TUB1::mCherry*<br>[Verified by PCR (of genomic DNA isolated from the yeast transformants) with primers JW2009 and JW1623, producing 1,125 bp PCR product]				
CN0058	5'- TTGCTTCCCAGTTAAGCTCACCAAAACACAAATACTCACTACAATACAAT**ATG**AGTAAAGGAGAAGAACTTTTCACTG -3'	Forward; having 50 nt upstream of ***ASN2*** start codon + ATG (bold) + ***GFP*** coding sequence (nt4-25)	JW1995	2,536 bp
JW1995	5'- CCGCATTTCTTGGTTCACTCGTCAATTATAAGAATACGATTGCGCTCGTA ATCGATGAATTCGAGCTCG -3'	Reverse; with 50 nt downstream of ***ASN2*** stop codon and homologous sequence to plasmid (underlined)	CN0058	
**Mutagenic primers for introducing R344A mutation to *ASN1* coding sequence**<br>**Plasmid template:** pFA6a-ASN1-GFP-NLS-kanMX6<br>**Resulting plasmid:** pFA6a-asn1(R344A)-GFP-NLS-kanMX6				
JW2259	5'- GACGTTACCACTATCGCAGCTTCCACTCCAATG -3'	Forward; to make R344A mutation (underlined) in ***ASN1***	JW2260	6,617 bp
JW2260	5'- GTAAGTTTCCAAATGGTAGATCAC -3'	Reverse; ***ASN1*** and ***ASN2*** share the same sequence	JW2259	
**Mutagenic primers for introducing R343A mutation to *ASN2* coding sequence**<br>**Plasmid template:** pFA6a-ASN2-GFP-NLS-kanMX6<br>**Resulting plasmid:** pFA6a-asn2(R343A)-GFP-NLS-kanMX6				
CN0056	5'- GACGTTACCACTATCGCAGCTTCTACACCAATG -3'	Forward; to make R343A mutation (underlined) in ***ASN2***	JW2260	6,617 bp
JW2260	5'- GTAAGTTTCCAAATGGTAGATCAC -3'	Reverse; ***ASN1*** and ***ASN2*** share the same sequence	CN0056	
**Primers for making DNA cassette *asn1(R344A)::GFP::NLS; kanR* (for transforming yeast *TUB1::mCherry*)**<br>**Plasmid template:** pFA6a-asn1(R344A)-GFP-NLS-kanMX6				

Primer	Sequence	Description	Used with	Product size
**Resulting yeast strain:** *asn1(R344A)::GFP::NLS TUB1::mCherry*				
JW2038	5'- CGCATTCCTTCCACCCCAATAG -3'	Forward; located at nt601-622 of ***ASN1*** coding sequence	JW1993	3,614 bp
JW1993	5'- AAATATCTATAAGATTAATCCATAATTCTTTTTCTATTTTTTAATGTTATATCGATGAATTCGAGCTCG -3'	Reverse; with 50 nt downstream of ***ASN1*** stop codon and homologous sequence to plasmid (underlined)	JW2038	
**Primers for making DNA cassette *asn2(R343A)::GFP::NLS; kanR* (for transforming yeast *TUB1::mCherry*)**<br>**Plasmid template:** pFA6a-asn2(R343A)-GFP-NLS-kanMX6<br>**Resulting yeast strain:** *asn2(R343A)::GFP::NLS TUB1::mCherry*				
JW2040	5'- CGTATCCCATCCACCCCAGTTGAC -3'	Forward; located at nt601-624 of ***ASN2*** coding sequence	JW1995	3,614 bp
JW1995	5'- CCGCATTTCTTGGTTCACTCGTCAATTATAAGAATACGATTGCGCTCGTA ATCGATGAATTCGAGCTCG -3'	Reverse; with 50 nt downstream of ***ASN2*** stop codon and homologous sequence to plasmid (underlined)	JW2040	
**Primers for preparing PCR products (genomic DNA isolated from yeast transformants as PCR templates) for strain verification by DNA sequencing (Macrogen)**<br>**Verified strains:**<br>(1) *ASN1::GFP::NLS TUB1::mCherry*<br>(2) *asn1(R344A)::GFP::NLS TUB1::mCherry*				
JW2010	5'- CTGCCCACTCGAGATGACAAATA -3'	Forward; located 200 nt upstream of ***ASN1*** start codon	JW1623	2,829 bp (with *NLS*)
JW1623	5'- GCGACCTCATACTATACCTG -3'	Reverse; located 184 nt downstream of ***GFP*** coding sequence	JW2010	
**Primers for preparing PCR products (genomic DNA isolated from yeast transformants as PCR templates) for strain verification by DNA sequencing (Macrogen)**<br>**Verified strains:**<br>(1) *ASN2::GFP::NLS TUB1::mCherry*<br>(2) *asn2(R343A)::GFP::NLS TUB1::mCherry*				
JW2009	5'- CATTGACTCATGGCAAGATTTCTCC -3'	Forward; located 200 nt upstream of ***ASN2*** start codon	JW1623	2,829 bp (with *NLS*)
JW1623	5'- GCGACCTCATACTATACCTG -3'	Reverse; located 184 nt downstream of ***GFP*** coding sequence	JW2009	
**DNA sequencing primers**				
JW2010	5'- CTGCCCACTCGAGATGACAAATA -3'	Forward; located 200 nt upstream of ***ASN1*** start codon		
JW2038	5'- CGCATTCCTTCCACCCCAATAG -3'	Forward; located at nt601-622 of ***ASN1*** coding sequence		
JW2039	5'- ACATCGATCCAAATGAAAAGATG -3'	Forward; located at nt1301-1323 of ***ASN1*** coding sequence		
JW2009	5'- CATTGACTCATGGCAAGATTTCTCC -3'	Forward; located 200 nt upstream of ***ASN2*** start codon		
JW2040	5'- CGTATCCCATCCACCCCAGTTGAC -3'	Forward; located at nt601-624 of ***ASN2*** coding sequence		
JW2041	5'- TTGATCCAAATGAAAAGATGATCAAG -3'	Forward; located at nt1301-1326 of ***ASN2*** coding sequence		
JW1623	5'- GCGACCTCATACTATACCTG -3'	Reverse; located 184 nt downstream of ***GFP*** coding sequence		
