# Supplementary material for: Nuclear targeted Saccharomyces cerevisiae asparagine synthetases associate with the mitotic spindle regardless of their enzymatic activity
Source: PLoS One. 2020 Dec 21;15(12):e0243742. doi: 10.1371/journal.pone.0243742 (PMC7751962; doi:10.1371/journal.pone.0243742)
Supplement: S2 File — (PDF) [file pone.0243742.s006.pdf]

S2 File. The data of fluorescence intensity within the nucleus and the cytoplasm of yeast *P_{ASN2}::GFP::NLS TUB1::mCherry*

No.	Fluorescence Intensity Mean Value		Fluorescence Intensity Ratio (Nucleus: Cytoplasm)
	Nucleus	Cytoplasm	
1	26140.50	8914.00	2.93
2	19610.00	6491.63	3.02
3	15077.88	5488.29	2.75
4	16811.38	7655.00	2.20
5	19590.29	5574.13	3.51
6	16621.88	5993.38	2.77
7	13218.25	5738.13	2.30
8	16409.88	5938.29	2.76
9	23811.57	8212.86	2.90
10	14926.63	5637.14	2.65
11	12830.71	5614.86	2.29
12	18561.88	6246.86	2.97
13	15901.88	5262.75	3.02
14	14638.29	5915.00	2.47
15	16917.75	7210.00	2.35
16	12215.25	4935.13	2.48
17	16083.00	4918.50	3.27
18	21326.57	7449.88	2.86
19	12003.25	4620.14	2.60
20	20332.88	7355.25	2.76
21	11597.25	4567.57	2.54
22	10860.00	5082.63	2.14
23	15369.00	5727.13	2.68
24	14004.63	5134.88	2.73
25	22445.43	9470.88	2.37
26	13669.00	5135.50	2.66
27	22527.13	7437.75	3.03
28	16829.75	4097.13	4.11
29	18872.00	7122.13	2.65
30	17783.57	5342.50	3.33
31	12099.25	4504.75	2.69
32	13110.63	5604.43	2.34
33	20629.50	7831.88	2.63
34	13644.25	4704.75	2.90
35	25018.50	8927.75	2.80
36	20852.88	7057.88	2.95
37	23071.88	8442.88	2.73
38	22070.38	7808.00	2.83
39	22951.13	8826.50	2.60
40	15221.75	5381.25	2.83
41	23414.38	9977.38	2.35
42	20483.50	8222.13	2.49

No.	Fluorescence Intensity Mean Value		Fluorescence Intensity Ratio (Nucleus: Cytoplasm)
	Nucleus	Cytoplasm	
43	21447.00	7875.13	2.72
44	26181.50	7546.75	3.47
45	19165.38	7804.00	2.46
46	19318.63	7607.29	2.54
47	26105.86	9420.13	2.77
48	19062.00	7464.13	2.55
49	25239.88	7720.50	3.27
50	28511.88	9299.50	3.07
51	23683.25	9197.88	2.57
52	17096.25	7574.00	2.26
53	18047.00	8738.00	2.07
54	20637.38	7111.29	2.90
55	21353.71	8393.43	2.54
56	24467.50	7548.63	3.24
57	24052.13	9481.00	2.54
58	22048.29	8611.75	2.56
59	16410.13	7592.00	2.16
60	17069.43	7354.25	2.32
61	19637.00	5779.13	3.40
62	16686.75	6452.38	2.59
		**Average**	**2.73**
