# Supplementary material for: Nuclear targeted Saccharomyces cerevisiae asparagine synthetases associate with the mitotic spindle regardless of their enzymatic activity
Source: PLoS One. 2020 Dec 21;15(12):e0243742. doi: 10.1371/journal.pone.0243742 (PMC7751962; doi:10.1371/journal.pone.0243742)
Supplement: S3 File — (PDF) [file pone.0243742.s007.pdf]

S3 File. The data of Western blot quantification for S2 Fig.

Exp#1				
**Strain**	**Anti-GFP (sum)**	**Anti-Pgk1p**	**Ratio (Anti-GFP: Anti-Pgk1p)**	**Relative to *GFP::NLS* control**
ASN1::GFP::NLS	11713.41	9816.29	1.19	**0.96**
asn1(R344A)::GFP::NLS	7265.46	10507.53	0.69	**0.55**
P_{ASN1}***::GFP::NLS***	12541.18	10038.70	1.25	**1.00**
ASN2::GFP::NLS	8467.58	10708.46	0.79	**4.04**
asn2(R343A)::GFP::NLS	9144.58	13233.36	0.69	**3.53**
P_{ASN2}***::GFP::NLS***	3361.62	17190.89	0.20	**1.00**
**Exp#2**				
**Strain**	**Anti-GFP (sum)**	**Anti-Pgk1p**	**Ratio (Anti-GFP: Anti-Pgk1p)**	**Relative to *GFP::NLS* control**
ASN1::GFP::NLS	13283.48	19383.48	0.69	**0.56**
asn1(R344A)::GFP::NLS	12750.14	19950.21	0.64	**0.53**
P_{ASN1}***::GFP::NLS***	23252.45	19144.97	1.21	**1.00**
ASN2::GFP::NLS	12852.41	23883.33	0.54	**1.56**
asn2(R343A)::GFP::NLS	11519.24	24269.13	0.47	**1.38**
P_{ASN2}***::GFP::NLS***	7219.93	20933.33	0.34	**1.00**

Average of Two Independent Experiments	
**Strain**	**Relative to *GFP::NLS* control**
ASN1::GFP::NLS	**0.76**
asn1(R344A)::GFP::NLS	**0.54**
P_{ASN1}***::GFP::NLS***	**1.00**
ASN2::GFP::NLS	**2.80**
asn2(R343A)::GFP::NLS	**2.45**
P_{ASN2}***::GFP::NLS***	**1.00**
